# Immune characteristics analysis and construction of a four-gene prognostic signature for lung adenocarcinoma based on estrogen reactivity

**DOI:** 10.1186/s12885-023-11415-y

**Published:** 2023-10-31

**Authors:** Yangwei Wang, Tong Yu, Jiaping Chen, Rong Zhao, Mingxin Diao, Peiyuan Mei, Shiwen He, Wenlin Qiu, Guanchao Ye, Lijuan Jiang, Han Xiao, Yongde Liao

**Affiliations:** 1 Department of Thoracic Surgery, Union Hospital, Tongji Medical College, Huazhong University of Science and Technology, Wuhan, China; 2 Department of Rheumatology and Immunology, Union Hospital, Tongji Medical College, Huazhong University of Science and Technology, Wuhan, China

**Keywords:** Estrogen, Lung adenocarcinoma, Immune cell infiltration, Prognosis, Nomogram

## Abstract

Lung adenocarcinoma (LUAD) is a common type of malignant tumor with poor prognosis and high mortality. In our previous studies, we found that estrogen is an important risk factor for LUAD, and different estrogen statuses can predict different prognoses. Therefore, in this study, we constructed a prognostic signature related to estrogen reactivity to determine the relationship between different estrogen reactivities and prognosis. We downloaded the LUAD dataset from The Cancer Genome Atlas (TCGA) database, calculated the estrogen reactivity of each sample, and divided them into a high-estrogen reactivity group and a low-estrogen reactivity group. The difference in overall survival between the groups was significant. We also analyzed the status of immune cell infiltration and immune checkpoint expression between the groups. We analyzed the differential gene expression between the groups and screened four key prognostic factors by the least absolute shrinkage and selection operator (LASSO) regression and univariable and multivariable Cox regression. Based on the four genes, a risk signature was established. To a certain extent, the receiver operating characteristic (ROC) curve showed the predictive ability of the risk signature, which was further verified using the GSE31210 dataset. We also determined the role of estrogen in LUAD using an orthotopic mouse model. Additionally, we developed a predictive nomogram combining the risk signature with other clinical characteristics. In conclusion, our four-gene prognostic signature based on estrogen reactivity had prognostic value and can provide new insights into the development of treatment strategies for LUAD.

**Supplementary Information:**

The online version contains supplementary material available at 10.1186/s12885-023-11415-y.

## Introduction

Lung cancer is the most common malignancy and the leading cause of cancer-related death worldwide [1]. Non-small cell lung cancer (NSCLC) accounts for 85% of lung cancer and mainly comprises lung adenocarcinoma (LUAD) and lung squamous cell carcinoma (LUSC) [2]. NSCLC cases occurring in non-smokers are more common among women than among men [3, 4]. Additionally, female patients suffering from NSCLC were found to benefit less from the administration of immune checkpoint inhibitors than control groups [5–8]. In a study, premenopausal women had a worse prognosis than men and postmenopausal women, suggesting that estrogen adversely affects the prognosis of lung cancer in premenopausal women [9].

Estradiol (E2), estrone, and estriol are the three primary estrogens [10]. E2 strongly influences normal physiological processes and the progression of numerous diseases [11, 12]. The known estrogen receptors include estrogen receptor α (ERα), estrogen receptor β (ERβ), and an orphan G-protein-coupled receptor (GPER) [13]. ERβ is highly expressed in the NSCLC tumors of both men and women, while the expression of ERα is low [14]. GPER expression in lung cancer cells and tumors is higher than that in normal lung tissues [15]. Estrogen acts by binding to these receptors [10]. Although the effects of estrogen on NSCLC have been extensively investigated, several issues remain unresolved [16–21]. In our previous studies, we found that estrogen can promote NSCLC progression, metastasis, and tyrosine kinase inhibitor (TKI) resistance [22–24]. However, these studies were limited to a single biomarker or small sample size, and the conclusions lacked accuracy and reliability. Therefore, determining the role of estrogen in lung cancer from different perspectives is necessary. A study based on bioinformatics analysis identified several differentially expressed estrogen signaling pathway genes between tumor tissue and para-cancerous tissue [17]. Three genes were found to be related to the prognosis of lung cancer; however, the study did not analyze the correlations between estrogen reactivity and prognosis [17].

In this study, we analyzed differentially expressed genes (DEGs) and immune cell infiltration between different estrogen reactivity groups in LUAD using bioinformatics methods. Then, we identified key prognostic genes and constructed a risk signature to predict the prognosis of LUAD patients by LASSO regression and univariable and multivariable Cox regression [25]. We performed in vivo experiments to further investigate the role of estrogen in the progression of LUAD. Finally, we developed a nomogram to predict the overall survival (OS) of LUAD patients.

## Materials and methods

### Data download

The gene transcriptome profiling data and the corresponding clinical data on LUAD were downloaded from The Cancer Genome Atlas (TCGA) database (TCGA-LUAD, https://portal.gdc.cancer.gov) and the Gene Expression Omnibus (GEO) database (GSE31210, https://www.ncbi.nlm.nih.gov/gds) [26, 27]. Only LUAD patients with intact survival time and status were included in this study. Samples from the TCGA database were used as the training cohort, and the GSE31210 dataset [28] was used as the validation cohort. The clinical information of the patients in the two cohorts is presented in Table 1. Estrogen-related gene sets were collected from the hallmark gene sets in the Molecular Signatures Database (https://www.gsea-msigdb.org/), which included Hallmark estrogen response early and Hallmark estrogen response late [29].

**Table 1:** Clinical characteristics of LUAD patients from TCGA and GEO databases

	TCGA-LUAD N = 509	GSE31210 N = 226
Vital status		
Alive	326 (64%)	191 (85%)
Dead	183 (36%)	35 (15%)
**Recurrence**		
Yes		64 (28%)
No		162 (72%)
**Gender**		
Female	274 (54%)	121 (54%)
Male	235 (46%)	105 (46%)
**Age (missing value: 10)**		
>=65	277 (54%)	62 (27%)
< 65	222 (44%)	164 (73%)
**Clinical stage**		
Stage I	273 (54%)	168 (74%)
Stage II	122 (24%)	58 (26%)
Stage III	81 (16%)	
Stage IV	25 (5%)	
**T stage**		
T1	171 (34%)	
T2	272 (53%)	
T3	45 (9%)	
T4	21 (4%)	
**N stage**		
N0	329 (65%)	
N+	177 (35%)	
**M stage**		
M0	481 (95%)	
M+	24 (5%)	
**Smoking history**		
Yes	359 (71%)	111 (49%)
No	150 (29%)	115 (51%)
**Malignancy history**		
Yes	82 (16%)	
No	427 (84%)	

### Classification of samples based on estrogen reactivity

All analyses were performed in the R software (version 4.2.2; https://www.r-project.org/). The normalized enrichment score (NES) of each member in the hallmark gene sets was calculated by the gene set variation analysis (GSVA) method using the “GSVA” package in the TCGA-LUAD cohort [30]. Samples with an NES above the median for the Hallmark estrogen response early and Hallmark estrogen response late gene sets were placed in the high-estrogen reactivity group, and samples with an NES below the median in the two gene sets were placed in the low-estrogen reactivity group. The Kaplan-Meier (K-M) survival analysis was performed using the “survival” and “survminer” packages to evaluate the prognostic differences between the groups. The heatmap of the results of GSVA was visualized using the “pheatmap” package and the differences in the estrogen-related gene set cluster were analyzed using the “ggpurb” package.

### Differential expression and enrichment analysis

All analyses were performed in the R software (version 4.2.2; https://www.r-project.org/). False discovery rate (FDR) adjustment was used when multiple testing adjustment was applied. The DEGs between two estrogen reactivity groups in the TCGA-LUAD cohort were identified using the “DESeq2” packages based on the thresholds of adjusted p-value < 0.05 and |log2-fold change (FC)| > 1 [31]. The DEGs were visualized by the volcano plot using the “ggplot2” package. Then, Gene Set Enrichment Analysis (GSEA) based on hallmark gene sets, Gene Ontology (GO) enrichment analysis, and Kyoto Encyclopedia of Genes and Genomes (KEGG) enrichment analysis were performed using the “clusterProfiler” package [32, 33]. The enrichment was considered to be statistically significant at an adjusted p-value < 0.05. The enrichment results were visualized using the “ggplot2” and “enrichplot” packages.

### Immune cell infiltration analysis

All analyses were performed in the R software (version 4.2.2; https://www.r-project.org/). The CIBERSORT algorithm was used to analyze the abundance of 22 types of tumor-infiltrating immune cells in all TCGA-LUAD samples [34, 35]. The results were visualized using a bar plot. Then, a correlation heatmap was constructed to determine the correlation of 22 types of immune cells using the “corrplot” package, and the “ggpurb” package was used to compare and visualize the abundance of each type of immune cell between the estrogen reactivity groups. The expression of key immune checkpoints, including PD-1, PD-L1, CTLA4, LAG3, and TIGIT, were also compared between the groups.

### Antibodies and reagents

Anti-ESR2 (Cat. No. 14,007–1-AP) and anti-ESR1 (Cat. No. 21,244–1-AP) antibodies were purchased from Proteintech (CA, USA). Estradiol (Cat. No. HY-B0141) and D-Luciferin sodium (Cat. No. HY-12,591) were purchased from MCE (Shanghai, China).

### Cell lines and transfection

Lewis Lung Carcinoma (LLC) cells were obtained from the American Type Culture Collection (ATCC). The LLC cells were grown in complete DMEM (DMEM, 10% FBS (GIBCO). All cells were cultured in a humidified incubator at 37℃ with 5% CO2. Lentiviral transfection expressing firefly luciferase reporter was obtained from Genechem Co., Ltd. (Shanghai, China). Transfection was performed following the manufacturer’s protocol. The cells with stable transfection were selected with puromycin. The Firefly Luciferase expression levels in LLC cells were detected using the Dual-Luciferase® Reporter (DLR™) Assay System (Promega).

### Animal experiments

A total of 9 female C57BL/6 mice (seven weeks old) were obtained from Beijing Vital River Laboratory Animal Technology Co. Ltd. They were randomly divided into three groups (n = 3 per group). One group served as the control, another group underwent ovarian removal, and a third group underwent ovarian removal and received E2. Ovariectomy was performed following the methods described in another study [36]. Ovariectomized (OVX) mice were intraperitoneally injected with PBS or E2 (100 µg/kg) every day. The LLC cells (1 × 10^6^) expressing firefly luciferase were injected directly into the left lung (in 50 µL of 1:1 mix of PBS and Matrigel (BD Biosciences)) three days after surgery [37]. After 12 days, bioluminescence imaging experiments were performed using a Bruker In-Vivo MS FX Pro small animal optical imaging system.

### Construction and validation of a novel prognostic signature

All analyses were performed in the R software (version 4.2.2; https://www.r-project.org/). The least absolute shrinkage and selection operator (LASSO) regression algorithm with 10-fold cross-validation was used to narrow down the prognosis-related DEGs using the “glmnet” package [38]. Then univariable and multivariable Cox regression analyses were conducted to determine the signature genes and calculate the corresponding regression coefficients. The K-M survival analysis was performed to further evaluate the prognostic value of each signature gene. The risk score was calculated for the TCGA-LUAD cohort using the following formula:

$$risk\,score\, = \,\sum\limits_{i = 1}^\infty {Coefficient(mRNAi)\, \times \,Expression(mRNAi)}$$

The TCGA-LUAD patients were divided into the low-risk group and the high-risk group based on the median risk score. The K-M curve was generated to compare OS between the low-risk and high-risk groups. The time-dependent receiver operating characteristic (ROC) was evaluated to estimate the predictive value of signature for OS using the “timeROC” package. To validate the applicability of this risk signature in different populations, the TCGA-LUAD patients were divided into different subgroups, and univariable Cox regression was conducted. The risk signature was also applied to the validation cohort GSE31210 to further assess its prognostic value for recurrence-free survival (RFS) and OS.

### Immunofluorescence staining

Cells were fixed with 4% paraformaldehyde in 1x PBS, and then, permeabilized in 1x PBS with 5% BSA and 0.4% Triton X-100. Fixed cells were incubated with the primary antibodies anti-ESR2 (Dilution: 1:200) or anti-ESR1 (Dilution: 1:200) overnight at 4 °C, washed thrice in 1x PBS and then incubated with the secondary antibody (Goat anti-rabbit Alexa Fluor488-conjugated, dilution: 1:500) for 1 h at room temperature. The nucleus was stained with DAPI. Images were captured using the Nikon A1 Confocal microscope (Nikon, Tokyo, Japan).

### Development of a nomogram

To further evaluate the prognostic value of our risk signature and other clinical characteristics, including gender, age, tumor (T) stage, nodal (N) stage, metastatic (M) stage, clinical stage, smoking history, and malignancy history, univariable and multivariable Cox regression analyses were conducted. Then, a nomogram was constructed based on the T stage, N stage, clinical stage, and risk score using the “rms” package. The Concordance index (C-index) was calculated to evaluate the discriminative ability of the nomogram and calibration curves were drawn to show the consistency between the predicted one-year, three-year, and five-year endpoint events and the authentic outcomes.

### Statistical analyses

All analyses were performed in the R software (version 4.2.2; https://www.r-project.org/) and the GraphPad Prism 8.0 software. Correlations were calculated using Spearman’s correlation analysis. The differences between the groups were assessed by performing the Wilcoxon rank sum test. Two-way ANOVA was performed to determine the differences in body weight over time between animal groups. The differences in OS and RFS were assessed by performing a Kaplan-Meier survival analysis and the log-rank test. FDR adjustment was used when multiple testing adjustment was applied. All statistical tests were two-tailed, and the differences between groups were considered to be statistically significant at p < 0.05 (*p < 0.05, **p < 0.01, ***p < 0.001, ****p < 0.0001).

## Results

### Identification of estrogen reactivity subtypes in LUAD

**Fig. 1:**
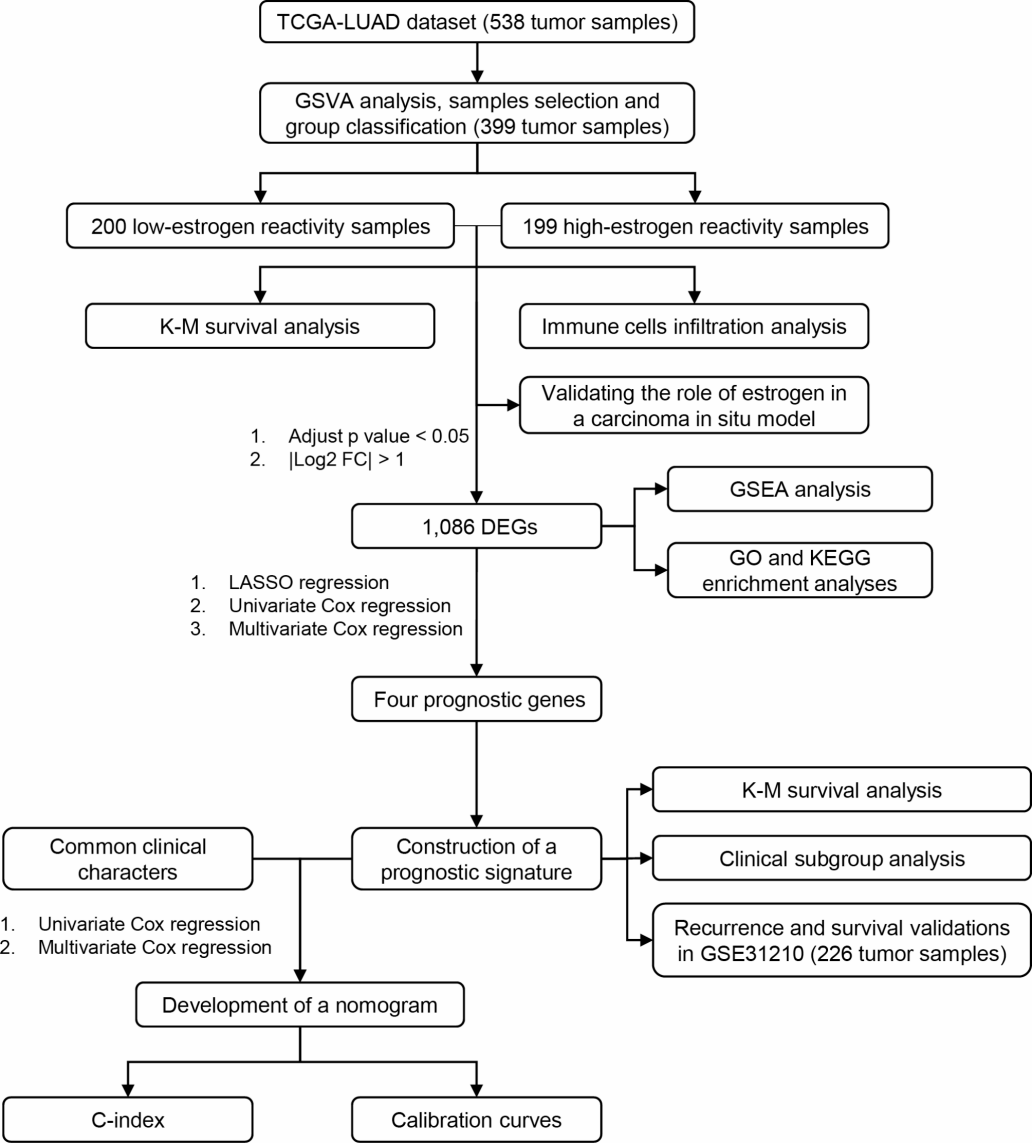
Flowchart of the present study

**Fig. 2:**
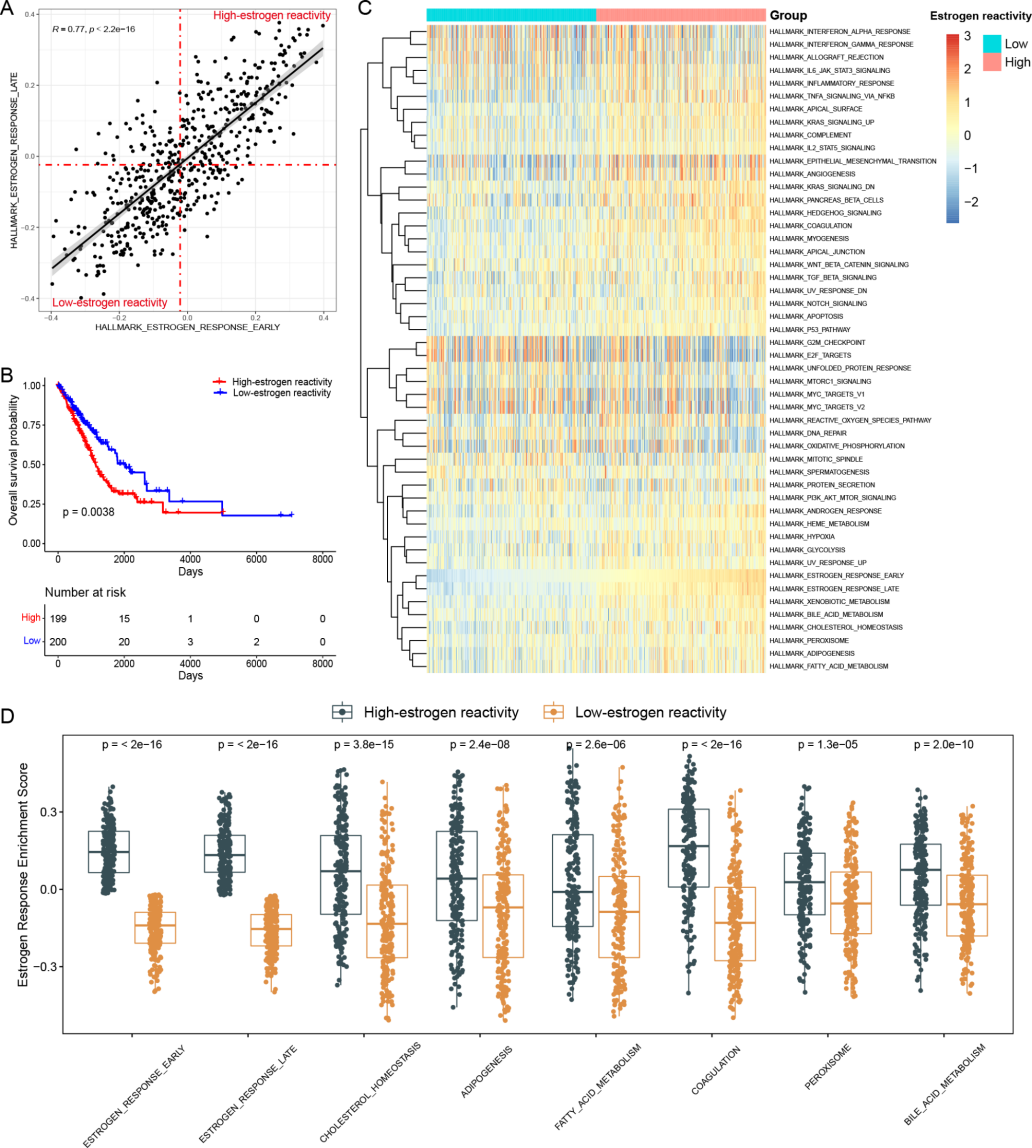
Identification of estrogen response patterns in the TCGA-LUAD cohort. (**A**) The correlation of estrogen response early scores and estrogen response late scores was calculated by GSVA. (**B**) K-M curve of OS between the estrogen reactivity groups. (**C**) Heatmap of GSVA scores in two groups. (**D**) Comparison of GSVA scores of 8 gene sets in estrogen response clusters between the groups

The flowchart of our study is shown in Fig. 1. Using hallmark gene sets as a reference, we obtained the normalized enrichment score (NES) of each tumor sample by performing GSVA using the data from the TCGA-LUAD cohort. The results of the correlation analysis showed that the NESs of the two estrogen-related gene sets (Hallmark estrogen response early and Hallmark estrogen response late) were strongly correlated (R = 0.77, p < 0.001) (Fig. 2A). Estrogen activates both of these estrogen-related gene sets at the same time. Based on the NESs of these two estrogen-related gene sets, the TCGA-LUAD samples were divided into two groups; 200 samples in the low-estrogen reactivity group and 199 samples in the high-estrogen reactivity group (Fig. 2A). The K-M curve showed that the difference in OS between the groups was significant (p = 0.0038) (Fig. 2B). The NESs of all 50 gene sets were displayed in the heatmap, from which six lipid metabolism-related gene sets were identified. Hallmark xenobiotic metabolism, Hallmark bile acid metabolism, Hallmark cholesterol homeostasis, Hallmark peroxisome, Hallmark adipogenesis, and Hallmark fatty acid metabolism were grouped into one cluster with the two estrogen-related gene sets (Fig. 2C). By comparing the NESs of these six gene sets, we found that their reactivities were significantly upregulated in the high-estrogen reactivity group (Fig. 2D). These results were consistent with the finding that estrogen promotes lipid metabolism [39].

### Identification of DEGs and functional enrichment

**Fig. 3:**
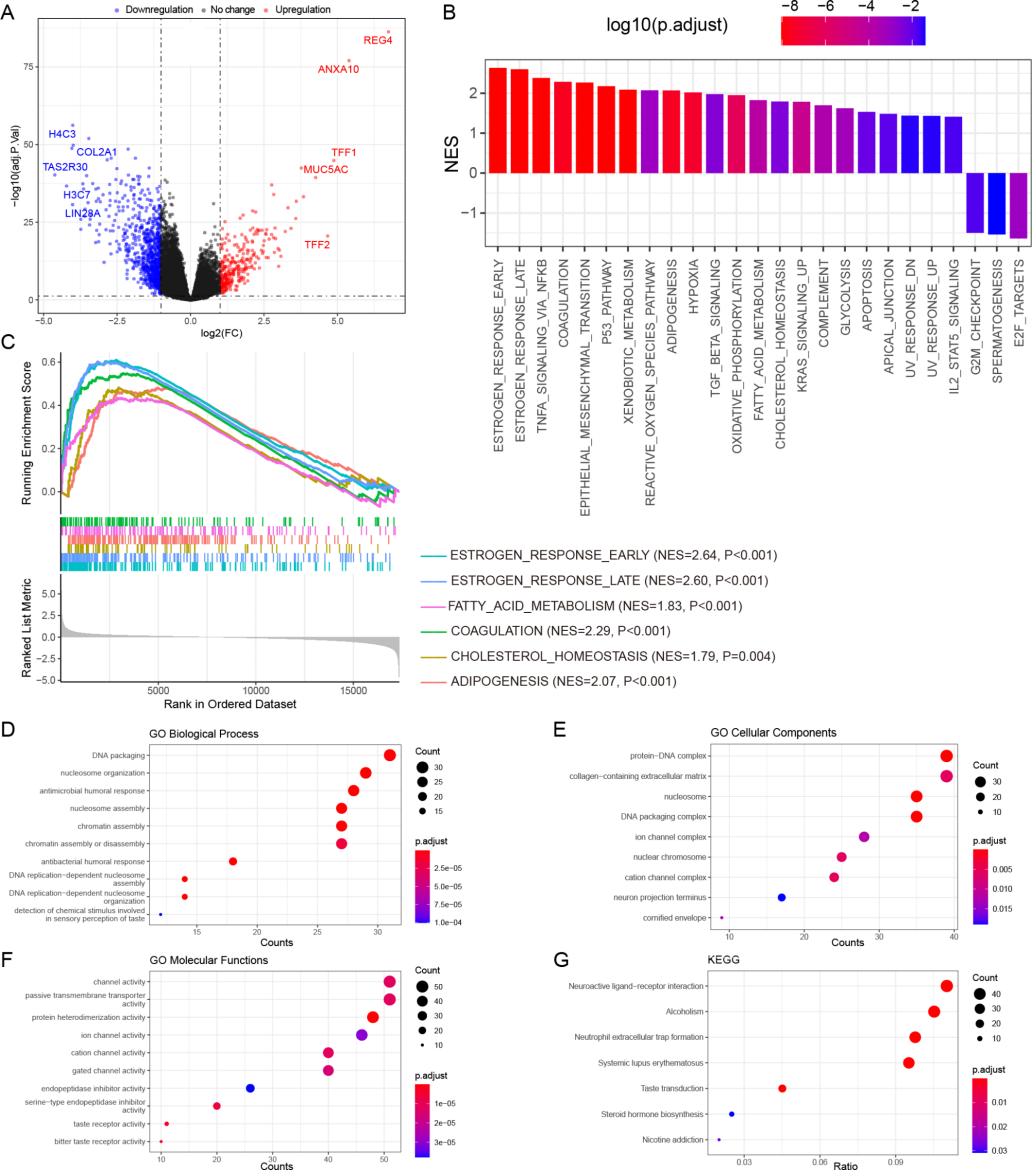
Identification of DEGs and functional enrichment analyses. (**A**) Volcano plot of the gene expression changes (the red plots represented upregulated genes and the blue plots represented downregulated genes in the high-estrogen reactivity group). (**B-C**) GSEA analysis of the groups. (**D-F**) GO analysis of the DEGs. (**G**) KEGG analysis of the DEGs.

The DEGs between the groups were identified and 1,086 DEGs were obtained. Compared to the expression of the genes in the low-estrogen reactivity group, 795 genes were downregulated and 251 genes were upregulated in the high-estrogen reactivity group. The distribution of these DEGs is shown in the volcano plot (Fig. 3A). Taking the above 50 gene sets as a reference, GSEA was performed. In total, 25 gene sets with significant alterations between the low-estrogen reactivity group and the high-estrogen reactivity group were obtained, among which the two estrogen-related gene sets had the highest enrichment scores (Fig. 3B). Four of the above six lipid metabolism-related gene sets were significantly upregulated in the high-estrogen reactivity group, including Fatty acid metabolism (p = 9.18e-05), Coagulation (p = 2.12e-08), Cholesterol homeostasis (p = 0.004), and Adipogenesis (p = 2.57e-08) (Fig. 3C).

Next, we performed the GO and KEGG enrichment analyses on the 1,086 DEGs. After estrogen binds to the receptor, it immediately enters the nucleus and initiates gene transcription. Many terms in the results of the GO and KEGG enrichment analyses were related to DNA transcription enhancement, and these terms were also involved in tumor progression. In the GO analysis, the top biological process (BP) terms were DNA packaging, nucleosome organization, antimicrobial humoral response, and nucleosome assembly (Fig. 3D). The terms related to the cellular components (CC) included protein-DNA complex, collagen-containing extracellular matrix, nucleosome, and DNA package complex (Fig. 3E). Regarding molecular functions (MF), the DEGs were mainly enriched in channel activity, passive transmembrane transporter activity, protein heterodimerization activity, and ion channel activity (Fig. 3F). The enriched KEGG pathways mainly included neuroactive ligand-receptor interaction, alcoholism, and neutrophil extracellular trap formation (Fig. 3G). More detailed enrichment results are presented in Supplementary Tables 1–3.

### Immune cell infiltration analysis

**Fig. 4:**
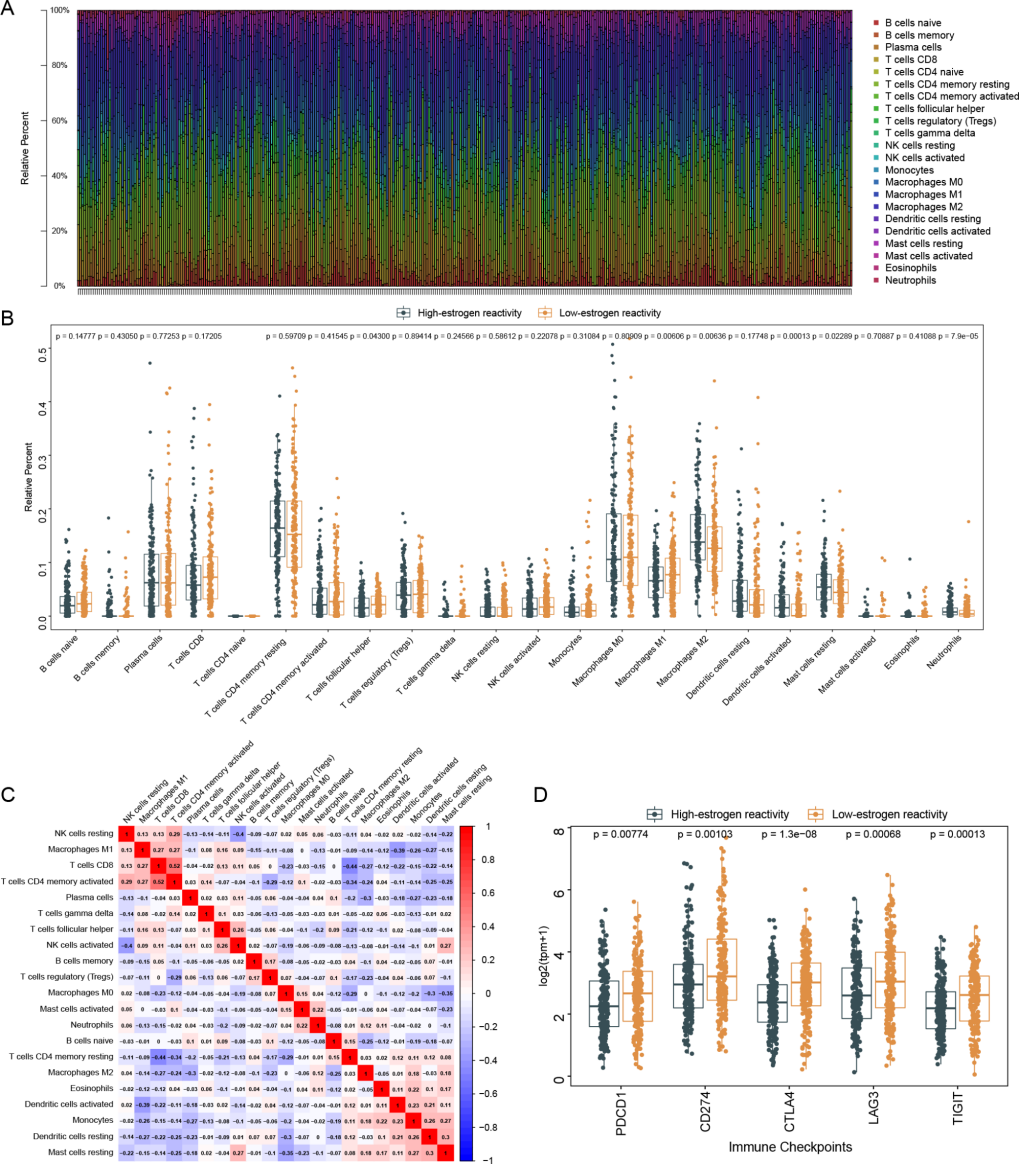
Immune cell infiltration analysis and expression of immune checkpoints. (**A**) The abundance of 22 types of immune cells in each sample. (**B**) Comparison of levels of 22 types of immune cells between the groups. (**C**) The correlations of levels of every two types of immune cells. (**D**) Comparison of expression of 5 immune checkpoints between the groups

Many immune cells were found to infiltrate the tumor tissue, and the CIBERSORT algorithm was applied to obtain the abundance of 22 types of immune cells in each sample. The bar plot shows the proportion of the 22 types of immune cells in each sample of the TCGA-LUAD cohort (Fig. 4A). The distribution of the proportion of each immune cell type in the two estrogen reactivity groups was compared. Among them, the abundance of M2 macrophages (p = 0.00636), activated dendritic cells (p = 0.00013), and neutrophils (p = 7.9e-05) increased, while the abundance of M1 macrophages (p = 0.00606) decreased in the high-estrogen reactivity group (Fig. 4B). The correlation heatmap of the 22 types of immune cells showed that activated memory CD4 T cells and CD8 T cells had the most significant positive correlations (r = 0.52) (Fig. 4C). Immune checkpoints are important for predicting the response to immunotherapy in LUAD. A study has shown that high expression of immune checkpoints might predict a better response to immunotherapy [40]. Therefore, we analyzed the expressions of some key immune checkpoints between the groups. The results showed that the expression of CTLA4 (p = 1.3e-08), PD-1 (p = 0.00774), PD-L1 (p = 0.00103), LAG3 (p = 0.00068), and TIGIT (p = 0.00013) was significantly lower in the high-estrogen response group compared to that in the low-estrogen response group (Fig. 4D).

### Estrogen promotes LUAD progression in vivo

**Fig. 5:**
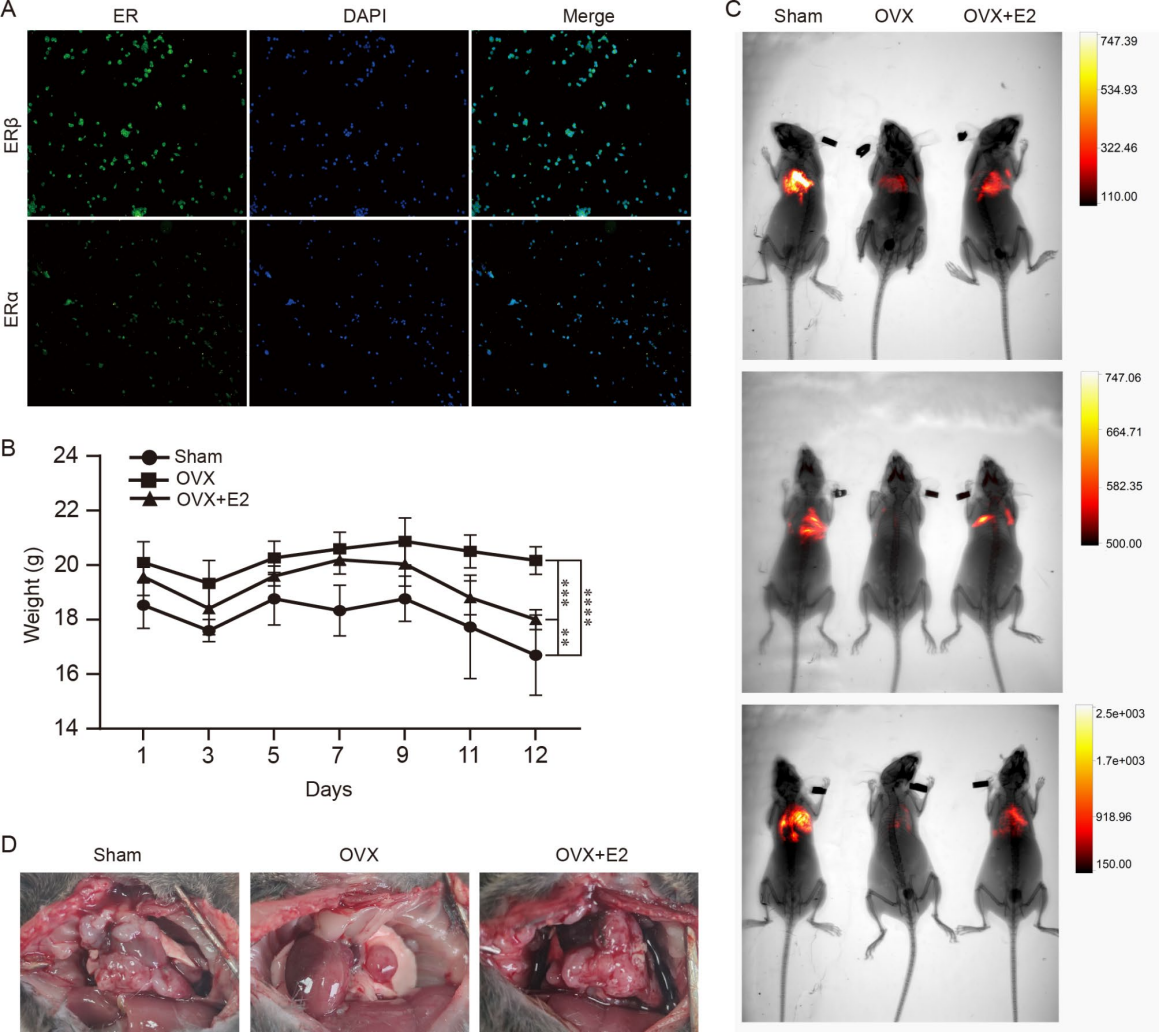
Validating the role of estrogen in an orthotopic mouse model. (**A**) The expression of ERα and ERβ in LLC cells expressing firefly luciferase was determined by immunofluorescence. (**B**) Changes in body weight over time after LLC injection (n = 3 per group). The two-way analysis of variance (ANOVA) was calculated using Prism 8 (GraphPad) (**p < 0.01, ***p < 0.001, ****p < 0.0001). Detailed data on body weight are shown in Supplementary Table 4. (**C**) Images of mice detected by a bioluminescence imaging system. (**D**) Representative thoracic anatomy images of mice

In another study, we showed that estrogen promotes NSCLC progression in immunodeficient mice [22]. Tumor growth is mainly influenced by the immune system [41]. Therefore, we selected immunocompetent C57BL/6 mice and administered in situ injections to construct a lung adenocarcinoma model. First, we confirmed the expression of ERα and ERβ in LLC cells by immunofluorescence (Fig. 5A). Then, nine female mice were divided equally among three groups, which included the control group, the OVX group, and the OVX + E2 group. The LLC cells were injected three days after ovariectomy, and the mice were weighed every two days. After injection, the mice lost weight rapidly. The mice in the control group were the fastest to lose weight, while those in the ovariectomy group showed the least change in weight (Fig. 5B). After 12 days, the bioluminescence imaging results showed that the size of the tumor decreased significantly after OVX, and the administration of estrogen abolished this tumor protection effect (Fig. 5C). Finally, after euthanizing the mice, they were dissected and photographed, and three representative pictures were selected for display (Fig. 5D).

### A novel prognostic signature for the prognostic prediction of LUAD

**Fig. 6:**
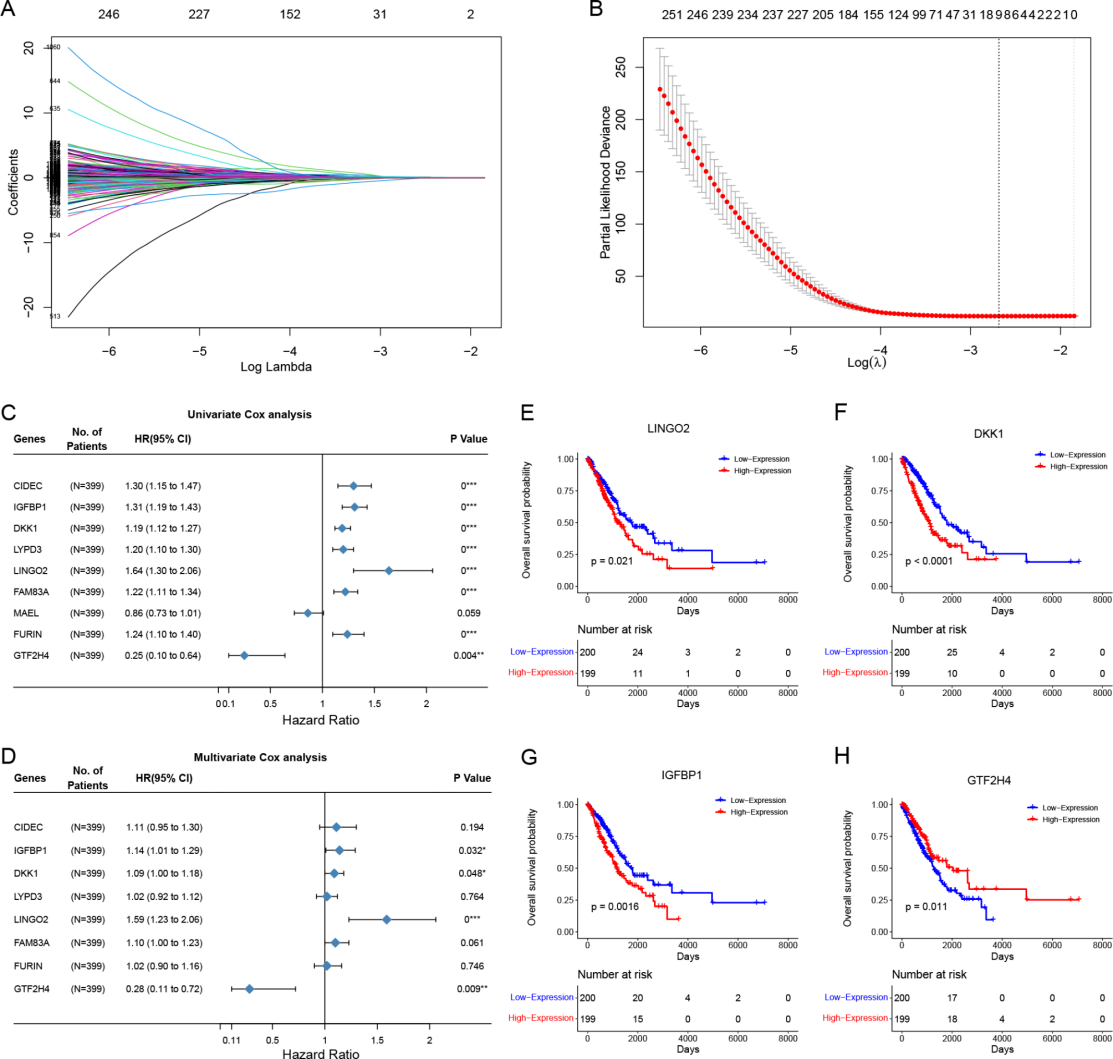
Selection of prognostic genes among DEGs. (**A-B**) LASSO regression analysis with 10-fold cross-validation. (**C-D**) univariable and multivariable Cox regression analyses of genes from LASSO regression. (**E-H**) K-M curves of OS between low- and high-expression groups of LINGO2, DKK1, IGFBP1, and GTF2H4

To further identify the key genes with a prognostic value among these DEGs, the LASSO regression based on the TCGA-LUAD cohort was conducted, and nine genes were obtained (CIDEC, IGFBP1, DKK1, LYPD3, LINGO2, FAM83A, MAEL, FURIN, and GTF2H4) (Fig. 6A, B). Then, univariable Cox and multivariable Cox analyses were performed for these nine genes, and four prognostic genes with p < 0.05, including IGFBP1, DKK1, LINGO2, and GTF2H4, were identified (Fig. 6 C, D). To further determine the prognostic values of the four genes, the TCGA-LUAD cohort was divided into two groups based on the median expression value of each gene. The K-M survival analysis was performed in each group, and the results showed that the OS for all genes differed significantly (IGFBP1 p = 0.0016; DKK1 p < 0.0001; LINGO2 p = 0.021; GTF2H4 p = 0.011). The high expression of IGFBP1, DKK1, and LINGO2 predicted a worse prognosis, while the high expression of GTF2H4 indicated a prognostic protective effect (Fig. 6E-H). Therefore, we selected these four genes to construct a risk signature with their expression levels and the corresponding coefficients from the multivariable Cox analysis results; the formula is as follows: risk score = (0.131 × IGFBP1) + (0.085 × DKK1) + (0.464 × LINGO2) + (-1.286 × GTF2H4).

**Fig. 7:**
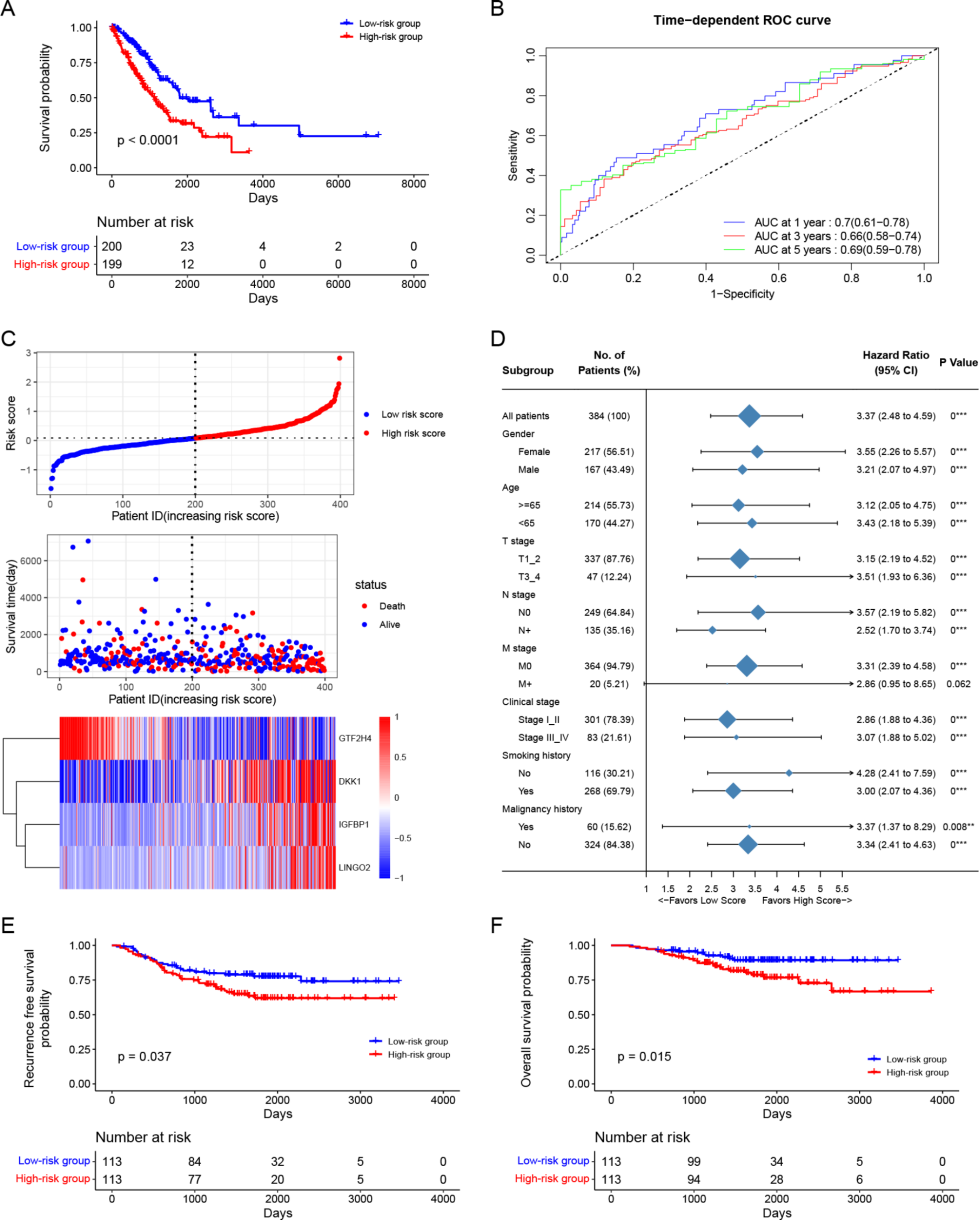
Construction and validation of a four-gene prognostic signature. (**A**) K-M curve of OS between low- and high-risk groups in the TCGA-LUAD cohort. (**B**) ROC curves of the risk score in the TCGA-LUAD cohort. (**C**) Risk plots in the TCGA-LUAD cohort. (**D**) Subgroups analysis in the TCGA-LUAD cohort. (**E**) Validation of recurrence-free survival between low- and high-risk groups in the GSE31210 cohort. (**F**) Validation of overall survival between low- and high-risk groups in the GSE31210 cohort

Based on the median value of the risk score, we divided the TCGA-LUAD cohort into the low-risk group and the high-risk group. The K-M curve showed that patients with lower risk scores lived longer than those with higher risk scores (Fig. 7A). The one-year, three-year, and five-year OS AUC values were 0.7, 0.66, and 0.69, respectively, which showed that the accuracy of risk signature needs to be improved (Fig. 7B). The risk score, survival status, and gene expression heatmap of the four prognostic genes are presented in the risk plots (Fig. 7C). Next, we further investigated whether the risk signature affected OS in the different clinical characteristic subgroups. We grouped the TCGA-LUAD cohort according to age, gender, T stage, N stage, M stage, clinical stage, smoking history, and malignancy history. We performed the univariable Cox analysis for each subgroup. The results showed that the risk signature had significant effects on survival for almost all subgroups (Fig. 7D). To validate the applicability of this risk signature, we downloaded the GSE31210 dataset from the GEO database, which contains data on OS and RFS. We used the same formula mentioned above to calculate the risk score for each patient and divided them into the low-risk group and the high-risk group based on the median risk score. The K-M curves showed that patients in the high-risk group had shorter RFS and OS than those in the low-risk group (Fig. 7E, F). To a certain extent, these results confirmed the applicability of our risk signature.

### Development of a nomogram

**Fig. 8:**
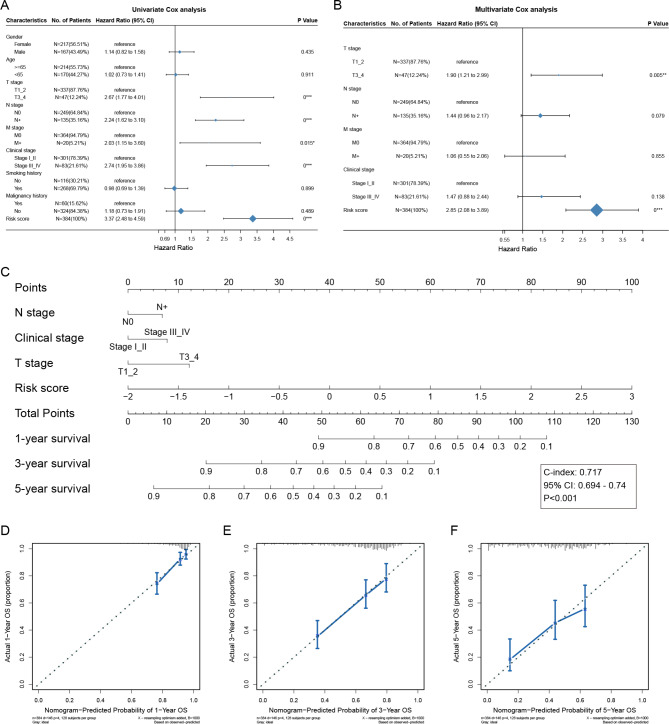
Development of a nomogram in the TCGA-LUAD cohort. (**A-B**) Univariable and multivariable Cox regression analyses of risk score and other clinical characteristics. (**C**) Development of a nomogram predicting the one-year, three-year, and five-year survival rates. (**D-F**) The calibration curves for predicting the one-year, three-year, and five-year survival

To determine whether the risk signature we constructed can serve as an independent prognostic factor, we performed univariable and multivariable Cox regression analyses using the risk score and other common clinical characteristics in the TCGA-LUAD cohort. The results of the univariable Cox regression analysis showed that the T stage, N stage, M stage, clinical stage, and risk score were strongly associated with OS (Fig. 8A). The results of the multivariable Cox regression analysis showed that the T stage and risk score were independent prognostic factors (Fig. 8B). Although the N stage and clinical stage were not significant as covariates, considering their clinical importance, they were also selected for constructing a nomogram to evaluate the probability of the one-year, three-year, and five-year OS (Fig. 8C). The C-index of the nomogram for predicting the OS was 0.717 (95% CI: 0.694–0.74, p < 0.001). The calibration curves of the one-year, three-year, and five-year OS showed good agreements between predicted survival and observed survival (Fig. 8D-F). These results suggested that our nomogram had good prognostic significance.

## Discussion

Many studies have investigated the relationship between estrogen and NSCLC [16–21]. Several studies have shown that different estrogen reactivities might affect the prognosis of NSCLC patients [42, 43]. In our previous study, we showed that estrogen is a pro-tumor factor for NSCLC [22]. However, models for accurately predicting the effects of estrogen reactivity on the OS of NSCLC patients are absent. The screening of prognostic biomarkers based on bioinformatics methods has been widely performed in studies on lung cancer. In this study, we used hallmark gene sets as the reference gene sets, and the samples in the TCGA-LUAD cohort were divided into the high-estrogen reactivity group and the low-estrogen reactivity group according to their estrogen reactivity scores based on the results of the GSVA. A risk signature was constructed based on DEGs between the groups. The results of the univariable and multivariable Cox regression analyses confirmed this risk signature as an independent prognostic factor in patients with LUAD, and this signature was validated using the GSE31210 dataset. We also showed the tumor-promoting effects of estrogen in vivo using an orthotopic mouse model of LUAD. Finally, we constructed a nomogram based on the risk signature and some clinical characteristics to predict the one-year, three-year, and five-year OS of the LUAD patients. The results showed that our nomogram was similar to the observed scenario.

Many immune cells were found to infiltrate tumor tissue, and these immune cells were involved in tumor metastasis, drug resistance, and immune escape [41]. Therefore, we analyzed the differences in the immune cell infiltration status between the groups with different estrogen reactivities. We found that the abundance of M2 macrophages increased while the abundance of M1 macrophages decreased in the high-estrogen reactivity group. In the tumor microenvironment, M1 macrophages have an antitumor effect, while M2 macrophages can promote immunosuppression [44]. These results suggested that estrogen might strongly influence tumor immune escape. We also studied the expression levels of five immune checkpoints, which were closely related to antitumor immunity [45]. Patients with high expression levels of these checkpoints respond better to immunotherapy. Our results showed that their expression levels were higher in the low-estrogen reactivity group, which suggested that immunotherapy had a greater effect on the patients in this group.

By performing differential expression analysis, we obtained 795 downregulated genes and 291 upregulated genes in the high-estrogen reactivity group. Among them, four key prognostic genes (LINGO2, DKK1, IGFBP1, and GTF2H4) were identified and used for constructing a risk signature. The results of the K-M survival analysis showed that our risk signature had excellent prognostic value. LINGO2 (Leucine Rich Repeat And Ig Domain Containing 2) was found by Carim-Todd et al. to be expressed in the early developmental stages of the central nervous system and also in the limbic system and cerebral cortex of adult tissues [46]. Studies on LINGO2 are limited and are mostly related to non-neoplastic diseases, such as Parkinson’s disease [47, 48]. Only one study on the molecular mechanism in gastric cancer found that it influences the progression of gastric cancer by altering gastric cancer initiation, stem cells, and cell motility tumorigenesis [49]. DKK1 (Dickkopf WNT Signaling Pathway Inhibitor 1) is a secreted protein that antagonizes the Wnt/b-catenin pathway. It regulates bone formation and affects the development and progression of bone metastases [50]. Several studies have indicated its role in the development, progression, and metastasis of tumors, including pancreatic ductal adenocarcinoma, breast cancer, ovarian cancer, cervical cancer, and endometrial cancer [51]. In deficient mismatch repair colorectal cancer, DKK1 can also attenuate the efficacy of immunotherapy by suppressing CD8 + T cells [52]. Based on the findings of studies on DKK1 in cellular and animal models, several clinical trials have been initiated to evaluate the safety and efficacy of anti-DKK-1 neutralizing antibodies in cancer [53]. One study used a panel of four genes (including DKK1) to predict the OS of LUAD [54]. The regulatory relationship between DKK1 and estrogen was also investigated. By preventing an increase in DKK1 levels, low physiological levels of E2 protect the hippocampal CA1 region against global cerebral ischemia [55]. IGFBP1 (Insulin Like Growth Factor Binding Protein 1) is the most prevalent IGFBP found in amniotic fluid and is typically expressed in the placenta, endometrium, and liver in a tissue-specific manner. After being secreted, IGFBP functions by interacting with IGFs [56]. Several studies have investigated its role as a biomarker in tumors such as gastrointestinal tumors and prostate cancer [57, 58]. IGFBP1 is specifically expressed in ovarian clear-cell adenocarcinoma [59]. In breast cancer cells, 4-OHT suppresses IGF-1 signaling due to the accumulation of extracellular IGFBP1, which is mediated by GPER1 and CREB [60]. The transcription factor II H (TFIIH) component GTF2H4 (also known as p52) is involved in nucleotide excision repair [61]. Studies on its role in tumors are limited. Overall survival was found to be strongly correlated with GTF2H4 SNPs in lung cancer [62]. Estrogen regulation of IGFBP1 and DKK1 has been reported in previous studies. In breast cancer cells, estrogen regulates IGFBP1 expression via GPER1 [59]. The expression of DKK1 in CD4 + and CD8 + T cells was increased in ovariectomized mice. No literature has reported the correlation between LINGO2 and GTF2H4 and estrogen [63]. Our findings suggested that the estrogen signaling pathway might affect the progression and prognosis of LUAD by regulating the expression of these four genes.

In this study, we divided LUAD patients into the high-estrogen reactivity group and the low-estrogen reactivity group, which had clinically important prognostic significance. Based on this grouping, a risk signature and a nomogram were constructed, which could effectively predict the prognosis of LUAD patients. In another study, we showed the pro-cancer effects of estrogen using a subcutaneous tumor model. In this study, we confirmed the effects using an orthotopic mouse model and obtained more reliable results. However, our study had some limitations that should be addressed in subsequent studies. First, our study was based on data collected from public databases, and choosing the median as a threshold to binarize variables might not be the best solution. Thus, our findings need to be validated by conducting large prospective clinical trials. Second, we analyzed the differences in immune cell infiltration between different estrogen reactivity groups, but these differences need to be verified experimentally, which we aim to perform in our next study. Third, the ROC curve and AUC values of the prognostic signature in the validation set are not ideal (Supplement Fig. 1A, B). It might be brought on by variations in clinical characteristics such as patient counts, tumor stages, and smoking histories. Finally, information on the effects of three of the four key prognostic genes on LUAD (i.e., except DKK1) investigated in this study is limited. Hence, further cell and animal experiments need to be performed to elucidate the functions of these genes.

## Conclusion

To summarize, our present and previous studies showed that estrogen adversely affects the prognosis of LUAD. Differences in estrogen reactivity can be used to predict the OS of LUAD patients. We constructed a prognostic risk signature and developed a points-scoring system to predict the OS of LUAD patients. Our study elucidated the specific mechanisms by which estrogen promotes lung adenocarcinoma progression and identified promising prognostic indicators and potential therapeutic targets for treating patients with LUAD.

### Electronic supplementary material

Below is the link to the electronic supplementary material.

Supplementary Material 1

Supplementary Material 2

Supplementary Material 3

Supplementary Material 4

Supplementary Material 5

## Data Availability

The datasets analyzed for this study can be found in the TCGA (TCGA-LUAD, https://portal.gdc.cancer.gov) and GEO (GSE31210, https://www.ncbi.nlm.nih.gov/gds) databases.

## Abbreviations

LUAD: Lung adenocarcinoma
TCGA: The Cancer Genome Atlas
LASSO: Least absolute shrinkage and selection operator
ROC: Receiver operating characteristic
NSCLC: Non-small cell lung cancer
LUSC: Lung squamous cell carcinoma
E2: Estradiol
ERα: Estrogen receptor α
ERβ: Estrogen Receptor β
GPER: G-protein-coupled receptor
DEGs: Differentially expressed genes
TKI: Tyrosine kinase inhibitor
OS: Overall survival
NES: Normalized enrichment score
GSVA: Gene set variation analysis
K-M: Kaplan-Meier
GO: Gene Ontology
KEGG: Kyoto Encyclopedia of Genes and Genomes
FDR: False discovery rate
LLC: Lewis Lung Carcinoma
OVX: Ovariectomized
RFS: Recurrence-free survival
C-index: Concordance index
ATCC: American Type Culture Collection
NES: Normalized enrichment score
BP: Biological process
MF: Molecular functions
LINGO2: Leucine Rich Repeat And Ig Domain Containing 2
DKK1: Dickkopf WNT Signaling Pathway Inhibitor 1
IGFBP1: Insulin Like Growth Factor Binding Protein 1
GTF2H4: general transcription factor IIH subunit 4
