# Supplementary material for: Immune characteristics analysis and construction of a four-gene prognostic signature for lung adenocarcinoma based on estrogen reactivity
Source: BMC Cancer. 2023 Oct 31;23:1047. doi: 10.1186/s12885-023-11415-y (PMC10619241; doi:10.1186/s12885-023-11415-y)
Supplement: Supplementary file 5 — Supplementary Material 5 [file 12885_2023_11415_MOESM5_ESM.pdf]

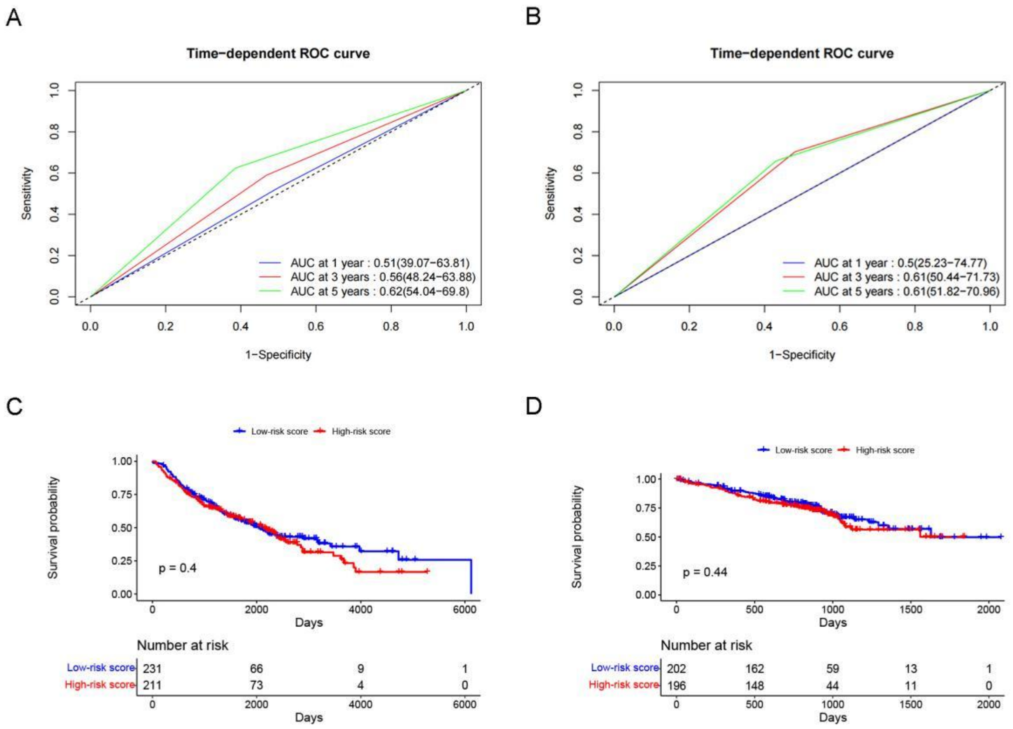

Supplement Figure 1. (A) ROC curves of risk score in the GSE31210 recurrence-free survival cohort. (B) ROC curves of risk score in the GSE31210 overall survival cohort. (C) Validation of overall survival between low- and high-risk groups in the GSE68465 cohort. (D) Validation of overall survival between low- and high-risk groups in the GSE72094 cohort.
